# FACTORS PROMOTING CONTINUED LIFELONG LEARNING: FOCUS ON THE PERSON-ENVIRONMENT FIT IN JAPAN

**DOI:** 10.1093/geroni/igz038.2450

**Published:** 2019-11-08

**Authors:** Chiharu Yasuzato, Keiko Katagiri

**Affiliations:** Graduate School of Human Development and Environment, Kobe University, Kobe, Hyogo, Japan

## Abstract

Lifelong learning of older people is categorized as social participation. Most studies on social participation have examined the motivation to start; however, those on continuing participation are few. This study aimed to identify the factors promoting continued lifelong learning among older adults, focusing on both personal and socio-relational factors. To do this, in-depth semi-structured interviews were conducted with 20 Japanese citizens, aged 60-75, with a learning experience of more than 3 years. Results showed that both personal and socio-relational factors matter. First, personal factors include older adults’ past learning experience, access to learning in the present, and work status. Past learning experience relates to their perception as students and their memories of learning during school age. Regarding accessibility, classes within walking distance, for example, would help in continuation of learning, especially for people with health problems. People may choose to become involved in learning activities after retiring from the workforce and they have time to spend. Second, the importance of socio-relational factors was evident in how family supported older people by accepting how important learning was for them and offering them rides to the classes. Within the class, they can share information about the happenings and activities in their community, become mentors, and stimulate each other. The instructor enhances their enthusiasm to learn and provides a comfortable learning space. Therefore, the findings of this study suggest that while there is no single condition, a person-environment fit promotes older adults continued learning.

